# Context-dependent regulation of SIX1 by ETS1 within EMT-associated transcriptional networks

**DOI:** 10.55730/1300-0152.2801

**Published:** 2026-03-23

**Authors:** İrem YALIM CAMCI

**Affiliations:** Department of Molecular Biology and Genetics, Faculty of Science, Gebze Technical University, Kocaeli, Turkiye

**Keywords:** EMT, ETS1, SIX1, HCC, transcriptional regulation, gene expression

## Abstract

**Background/aim:**

Epithelial-mesenchymal transition (EMT) is a fundamental process driving tumor plasticity, metastasis, and therapy resistance. Although E26 transformation-specific transcription factor 1 (ETS1) and Sine oculis homeobox homolog 1 (SIX1) are individually implicated in EMT-related transcriptional programs, the regulatory interplay and functional coordination of these proteins across tumor states remain unclear. Hence, this study aimed to investigate the mechanistic and clinical behavior of the ETS1–SIX1 axis, with a focus on hepatocellular carcinoma (HCC) and aggressive endothelial-like cancer phenotypes.

**Materials and methods:**

ETS1–SIX1 expression patterns were analyzed using real-time quantitative polymerase chain reaction in HCC-derived cell lines and the endothelial-like SK-HEP-1 cell line, whereas chromatin immunoprecipitation (ChIP) assays were performed in SK-HEP-1 cells to assess ETS1 binding to the SIX1 promoter. Clinical relevance was assessed using The Cancer Genome Atlas (TCGA) RNA-sequencing (RNA-seq) datasets (breast invasive carcinoma, colon adenocarcinoma, liver hepatocellular carcinoma (LIHC), and lung adenocarcinoma) with correlation and survival analyses, and validated using tumor cDNA panels and public transcriptomic data.

**Results:**

ChIP assays confirmed ETS1 binding to the SIX1 promoter. Expression analyses indicated an inverse relationship between ETS1 and SIX1 in HCC-derived cell lines, the endothelial-like SK-HEP-1 cell line, and tumor cDNA panels. Pan-cancer analyses showed decreased ETS1 and increased SIX1 expression in tumors, with stage-dependent heterogeneity. Elevated SIX1, but not ETS1, was associated with poorer overall survival, particularly in LIHC. Gene set enrichment analysis linked high-risk profiles to EMT, cell cycle, and immune-related pathways, while ETS1 expression decreased with increasing tumor grade.

**Conclusion:**

This study supported a model in which ETS1 negatively regulates SIX1 expression within liver cancer-associated contexts. Integrating mechanistic and clinical analyses, the findings suggest that the ETS1–SIX1 axis contributes to EMT-driven tumor plasticity and aggressive tumor behavior, with potential relevance for future therapeutic investigation.

## 1. Introduction

Epithelial-mesenchymal transition (EMT) is a fundamental biological process that allows epithelial and endothelial cells to acquire mesenchymal characteristics, including enhanced migratory capacity, invasiveness, and resistance to apoptosis (Thiery et al., 2009). EMT-related transcriptional reprogramming is increasingly recognized as a key driver of tumor progression, metastasis, and therapeutic resistance across multiple cancer types, including hepatocellular carcinomas (HCCs) (Lambert et al., 2018; Villanueva, 2019). EMT is not limited to epithelial tumor cells; it also occurs in endothelial and other highly plastic cell populations within the tumor microenvironment, where it facilitates invasion, vascular remodeling, and tumor dissemination (Kalluri and Weinberg, 2009).

Transcription factors (TFs) play a central role in orchestrating EMT-associated gene expression programs by integrating extracellular signals with chromatin-level regulation (Huang et al., 2022a). Among these, the E26 transformation-specific TF 1 (ETS1), a member of the ETS family, regulates genes involved in cell proliferation, differentiation, angiogenesis, and tissue remodeling (Garrett-Sinha, 2013; Dittmer, 2015). ETS1 has been implicated in aggressive tumor behavior and invasive, migratory phenotypes across diverse malignancies (Vishnoi et al., 2022; Wang et al., 2025). More recent evidence indicates that ETS1 coordinates hybrid EMT states that support metastatic dissemination and immune evasion (Ziman et al., 2025). ETS1 functions as a transcriptional activator for genes associated with EMT, including matrix metalloproteinase 2 (*MMP2*), *MMP7*, and *MMP9*, which facilitate extracellular matrix degradation and promote cellular invasion (Behrens et al., 2001; Chen et al., 2014). These characteristics establish ETS1 as a crucial regulator of transcriptional networks associated with EMT, rather than as a lineage-specific oncogenic factor.

Another TF closely linked to EMT and cellular plasticity is Sine oculis homeobox homolog 1 (SIX1), which is a developmental regulator that is largely silent in adult tissues but frequently reactivated in pathological contexts, particularly in cancer (Huang et al., 2022b; Balçık-Erçin et al., 2023). Aberrant *SIX1* expression promotes proliferation, invasion, EMT, metastasis, and therapeutic resistance via transcriptional reprogramming of mesenchymal and stemness-associated pathways (Ng et al., 2006; Yu et al., 2006; Iwanaga et al., 2012; Ono et al., 2012). *SIX1* transcription is regulated by EMT-inducing pathways such as transforming growth factor beta (TGF-β)/small mothers against decapentaplegic (SMAD) and Wingless-related integration site (Wnt)/β-catenin, which activate the *SIX1* promoter through SMAD- or β-catenin-dependent mechanisms (MacDonald et al., 2009; Xu et al., 2009; Sun et al., 2016). According to its functional role, increased SIX1 expression is associated with a negative prognosis across various tumor types (Micalizzi et al., 2009).

Despite the independent implication of ETS1 and SIX1 as key regulators, it was hypothesized herein that ETS1 may directly regulate the expression of SIX1 at the transcriptional level, based on its role in EMT-associated gene regulation and the established function of SIX1 as an EMT driver (Guo et al., 2025). To test this hypothesis, in silico analyses were performed to identify putative ETS1-binding motifs within the SIX1 promoter and examined the expression and regulatory interplay of ETS1 and SIX1 in highly plastic, EMT-prone cellular models. Rather than modelling hepatocyte-specific carcinogenesis, this study focused on the ETS1-mediated transcriptional control of EMT-associated programs in endothelial-like cells, revealing new transcriptional mechanisms that may operate broadly across tumor-associated cellular plasticity.

## 2. Materials and methods

### 2.1. Antibodies and materials

The following antibodies were used: ETS-1 (D808A) rabbit antibody (14069, Cell Signaling Technology, Danvers, MA, USA), rabbit IgG isotype control (ab37415; Abcam, Cambridge, UK), SIX1 rabbit polyclonal antibody (NBP1-844264; Novus Biologicals, Centennial, CO, USA), glyceraldehyde-3-phosphate dehydrogenase (GAPDH) (2118, Cell Signaling Technology), antimouse IgG, horseradish peroxidase (HRP)-linked antibody (7076S, Cell Signaling Technology), and anti-rabbit IgG, HRP-linked antibody (7074S, Cell Signaling Technology). Also used were the NucleoSpin RNA kit (740955.250; Macherey-Nagel, Düren, Germany), ProtoScript M-MuLV Taq real-time polymerase chain reaction (RT-PCR) kit (E6400S; New England Biolabs, Ipswich, MA, USA), TissueScan quantitative PCR (qPCR) Cancer Survey cDNA Array I (CSRT101; OriGene Technologies, Rockville, MD, USA), Polybrene (#TR-1003-G; Sigma-Aldrich Chemical Co., St. Louis, MO, USA), and puromycin (A1113802; Thermo Fisher Scientific, Waltham, MA, USA). The pLKO.1 plasmid vector (#8453; Addgene, Watertown, MA, USA) was used as a control, along with the human pLKO.1 shETS1 vectors (TRCN0000005591 and TRCN0000005592; Sigma-Aldrich Chemical Co.).

### 2.2. Cell lines and culture conditions

HCC-derived cell lines—including HepG2 and HUH7 (well-differentiated, epithelial-like), Hep3B and PLC/PRF/5 (epithelial morphology), and SK-HEP-1 (liver tumor-derived, exhibiting sinusoidal endothelial-like morphology)—were cultured in complete Dulbecco’s modified Eagle’s medium. Additionally, SNU182 and SNU398 (both HCC-derived, epithelial morphology) were cultured in complete Roswell Park Memorial Institute 1640 media. All media were supplemented with 10% fetal bovine serum and antibiotics. The cell medium was changed every two days. The cells were incubated in an incubator with 5% CO_2_ and 95% humidified air at 37 °C for 24 h.

### 2.3. Chromatin immunoprecipitation (ChIP)

ChIP assays were performed using standard protocols (Balcik-Ercin et al., 2018). Cells were crosslinked with formaldehyde and quenched with glycine (125 mM), followed by nuclear lysis in sodium dodecyl sulfate (SDS)-containing buffer. Chromatin was sheared by sonication using a Bioruptor Pico system (Diagenode, Denville, NJ, USA) under standard conditions. Ten percent of the lysate was retained as input, and the remaining chromatin was equally divided and immunoprecipitated with anti-ETS1 or rabbit IgG control antibodies. Immunocomplexes were washed sequentially with lithium chloride and tris(hydroxymethyl)aminomethane-ethylenediaminetetraacetic acid buffers, and DNA-protein complexes were eluted.

After RNase A and proteinase K treatment, crosslinks were reversed via incubation at 65 °C. ChIP DNA was purified using a QIAquick PCR purification kit (Qiagen GmbH, Hilden, Germany) and analyzed using ChIP–qPCR. Enrichment was assessed using 2 sets of primers targeting the SIX1 promoter, with the ZEB1 promoter used as a control region (Table 1).

### 2.4. Lentiviral particle production and cell transduction

Lentiviral particles expressing *shETS1* were prepared as previously described (Yalim-Camci et al., 2019) and used to achieve ETS1 silencing. For the transduction process, Sk-HEP-1 cells were seeded in 6-well plates. As the cells approached 80% confluency, they were infected with viral particles in the presence of 8 μg/mL of polybrene. The medium was replaced after 8–10 h, and the transduced Sk-HEP-1 cells were selected using puromycin (2.5 μg/mL) after 48 h. For shRNA-mediated knockdown experiments, cells transduced with the pLKO empty vector were used as the baseline control, in accordance with standard lentiviral knockdown protocols. Based on prior optimization studies and established literature (Stewart et al., 2003), no significant differences in basal ETS1 expression or global transcriptional profiles were expected between parental and pLKO-control cells.

### 2.5. Real-time qPCR (RT-qPCR)

Total RNA was isolated from shPLKO.1- and shETS1-SK-HEP-1 clones, as well as HCC cell lines, using the NucleoSpin RNA Kit (Macherey-Nagel). cDNA synthesis was performed using the ProtoScript M-MuLV RT-PCR Kit (New England Biolabs) according to the manufacturer’s instructions. RT-qPCR reactions were performed using Maxima SYBR Green qPCR Master Mix (Thermo Fisher Scientific) in a 20-μL reaction volume containing 50 ng of cDNA and 0.2 μM of primers. Amplification was performed under standard cycling conditions (95 °C initial denaturation, followed by 45 cycles at 95 °C, 60 °C, and 72 °C). ETS1 and SIX1 expression levels were normalized to GAPDH, and relative expression was calculated using the 2^–ΔΔCt method. Sequences of primers for cDNA amplification of ETS1 were 5′-GCATGTCGTTCCAAACTAACAC-3′ and 5′-GAAAGCCGTACACTTCTCTCTAC-3′, SIX1 primers were 5′-AAAGGGAAGGAGAACAAGGATAG-3′ and 5′-AGCCTACATGATTACTGGGATTT-3′. The primers for the normalizer GAPDH were 5′-GGCTGAGAACGGGAAGCTTGTCAT-3′ and 5′-CAGCCTTCTCCATGGTGGTGAAGA-3′.

### 2.6. Western blotting

Total protein lysates were prepared from ETS1-shRNA and control-shRNA SK-HEP-1 cell clones using SDS lysis buffer supplemented with a protease inhibitor cocktail (Roche, Basel, Switzerland). Protein concentrations were determined via bicinchoninic acid assay and quantified using a Qubit fluorometer (Thermo Fisher Scientific). Equal amounts of protein (30 μg) were separated by SDS-polyacrylamide gel electrophoresis and transferred onto polyvinylidene fluoride membranes (Millipore, Burlington, MA, USA). Membranes were blocked in Tris-buffered saline containing 5% nonfat dry milk and incubated overnight at 4 °C with primary antibodies against ETS1, SIX1, and GAPDH. This was followed by incubation with HRP-conjugated secondary antibodies for 1 h at room temperature. Protein bands were detected using a chemiluminescent substrate (SignalFire, Cell Signaling Technology) and visualized with a ChemiDoc XRS system (Bio-Rad Laboratories, Inc., Hercules, CA, USA) quantified by densitometric analysis using Image Lab software, with protein levels normalized to GAPDH.

### 2.7. Comparative expression of ETS1 and SIX1 in primary human cancers via cDNA microarray

The ETS1 and SIX1 mRNA expression levels were analyzed using RT-qPCR using 96-well cancer cDNA arrays (TissueScan; OriGene Technologies), including eight different tumor types: breast adenocarcinoma (BRCA), kidney carcinoma, colon adenocarcinoma (COAD), liver hepatocellular carcinoma (LIHC), thyroid carcinoma, lung adenocarcinoma (LUAD), ovarian adenocarcinoma, and prostate adenocarcinoma. cDNAs from nine tumors and three corresponding normal samples were included for each cancer type. The RT-qPCR assays were performed in duplicate, after which the SIX1 and ETS1 expression levels in the tumor tissues were calculated relative to those in the corresponding normal tissues using the ΔΔCt method (Livak and Schmittgen, 2001).

### 2.8. Bioinformatics analyses

RNA-sequencing (RNA-seq) raw count data and corresponding clinical annotations for The Cancer Genome Atlas (TCGA)-LIHC, TCGA-COAD, TCGA-BRCA, and TCGA-LUAD cohorts were obtained from TCGA using the TCGAbiolinks R package. Analyses were limited to primary solid tumors and available normal tissues. Raw counts were normalized using the counts per million (CPM) method implemented in EdgeR and log_2_ (CPM + 1) transformed. Differences in SIX1 and ETS1 expression between tumor and normal tissues were assessed using the Wilcoxon rank-sum test, while pathological stage-associated differences were analyzed using the Kruskal–Wallis test. For survival analyses, patients were stratified into high- and low-expression groups based on median expression levels. Overall survival was evaluated using Kaplan–Meier analysis with log-rank testing, and prognostic significance was assessed using univariate Cox proportional hazards regression. Gene set enrichment analysis (GSEA) was performed using the ClusterProfiler package (Subramanian et al., 2005), with genes preranked using Spearman correlation coefficients relative to SIX1 or ETS1 expression and tested against Hallmark gene sets from the Molecular Signatures Database. Pathways with a normalized enrichment score and false discovery rate < 0.05 were considered significant. Spearman correlation analyses were conducted to examine coexpression patterns between SIX1, ETS1, and selected proliferation and EMT markers. All analyses were performed in R 4.5.1, with p < 0.05 considered statistically significant.

### 2.9. Statistical analysis

All experiments were performed in triplicate on different days. Analyses were performed using GraphPad Prism 8. Quantitative data were expressed as the mean ± standard deviation (SD). p < 0.05 was considered statistically significant, with higher significance levels indicated as p < 0.05, **p < 0.01, and ***p < 0.001.

## 3. Results

### 3.1. ETS1 and SIX1 expression in HCC-derived cell lines and the endothelial-like SK-HEP-1 cell line

**Figure 1:**
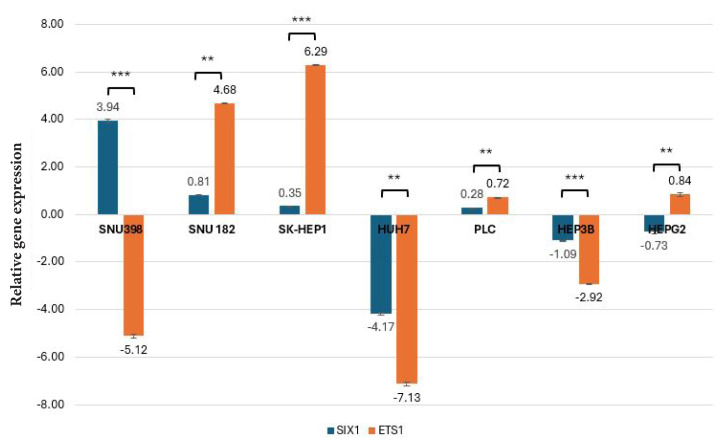
RT-qPCR analysis of ETS1 and SIX1 expression in HCC-derived cell lines and the endothelial-like SK-HEP-1 cell line. Relative gene expression levels were calculated using the ΔΔCt method. GAPDH was used as the housekeeping gene.

Initially, the gene expression profiles of ETS1 and SIX1 in the HCC-derived cell lines (SNU398, SNU182, HUH7, PLC, HEP3B, and HEPG2), and in the endothelial-like SK-HEP-1 cell line, were examined through RT-qPCR. Overall examination of the expression profile of all HCC cell lines demonstrated that expression levels of SIX1 were lower and more variable among the SNU398, SNU182, SK-HEP1, and HepG2 cell lines (Figure 1).

### 3.2. ETS1 binding to the SIX1 promoter

**Figure 2:**
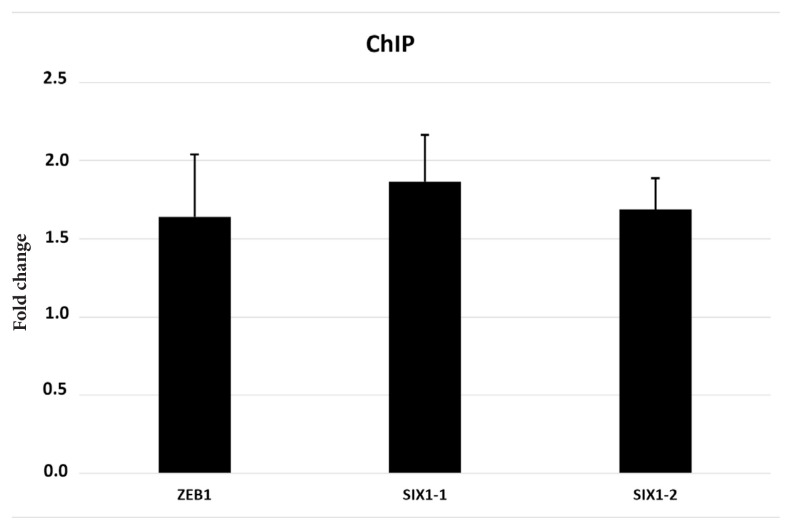
ChIP-qPCR assay demonstrating the binding of ETS1 to the SIX1 promoter region. Primers flanking the ETS1 binding motifs SIX1-1 (−386 bp from TSS) and SIX1-2 (-1697 bp from TSS) were used for ChIP-qPCR on DNA fragments immunoprecipitated with the ETS1 and isotype (control) antibodies. The concept of fold enrichment is presented as the ratio of the percentage of ETS1 antibody input to that of the control antibody. The ZEB1 gene regions were used as positive controls.

ChIP analysis was conducted in endothelial-like SK-HEP1, which exhibited high endogenous ETS1 expression. Previous studies have reported ETS1 binding sites in the promoter of ZEB1 (Sinh et al., 2017). ZEB1 was considered a positive control. The SIX1 ChIP-qPCR was performed with primers flanking ETS1 binding-consensus ETS-binding motif “GGAA/T” at −386 and −1697 bp upstream of the transcription start site of the SIX1 gene (Figure S1). ChIP-qPCR analysis revealed enrichment in the ZEB1 (1.5-fold), SIX1-1 (1.3-fold), and SIX1-2 (1.4-fold) gene regions (Figure 2).

### 3.3. ETS1 knockdown upregulation of SIX1 expression in endothelial-like SK-HEP-1 cells

**Figure 3:**
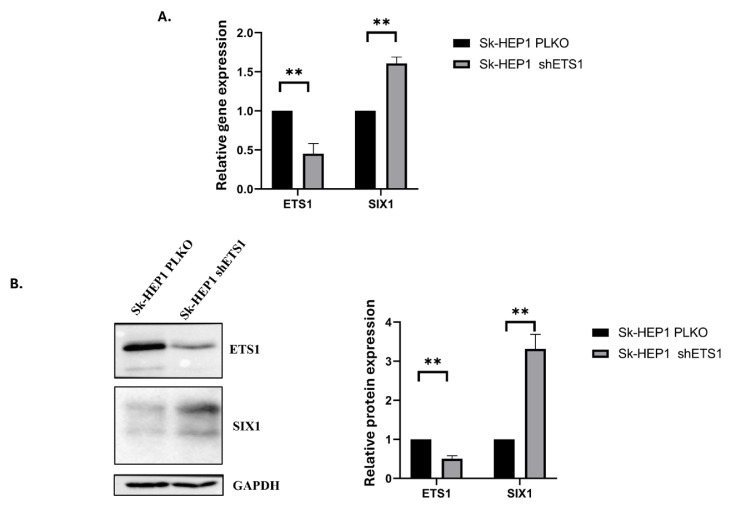
ETS1 regulates SIX1 expression in endothelial-like SK-HEP-1 cells. **A**. Relative mRNA expression levels of *ETS1* and *SIX1* in ETS1-knockdown SK-HEP-1 cells, as determined by RT-qPCR. **B**. Protein levels of ETS1 and SIX1 in shETS1 and shPLKO SK-HEP-1 cells, as assessed by Western blotting. GAPDH was used as a loading control.

In the subsequent experiment, the expression of ETS1-regulated SIX1 was examined by RT-qPCR using ETS1-shRNA-Sk-HEP1 (sh-ETS1) and using PLKO-shRNA-Sk-HEP1 (sh-PLKO) stable clones. In the context of ETS1 knockdown, a 64% decrease in *ETS1* expression was observed, accompanied by a 54% increase in *SIX1* expression (Figure 3A). Concurrent with the high transcript values, a significant rise in SIX1 protein levels was detected in the sh-ETS1 clone (Figure 3B).

### 3.4. Comparative expression analysis of ETS1 and SIX1 in tumor cDNA specimens

The increase of SIX1 in a clone where ETS1 was reduced, along with the notable results from the ChIP assay, warranted a closer look at how *SIX1* and *ETS1* are expressed in different cancers. Consequently, the expression levels of *SIX1* and *ETS1* were used in cancer cDNA arrays (comprising a total of eight different tissues, with each array containing three normal and nine tumor samples, respectively) for further analysis. A significant increase in *SIX1* expression was observed in the following tissues when compared to the control group: lung, liver, breast, colon, kidney, ovarian, and prostate tumors. A decrease in *SIX1* expression was observed in thyroid cancer cases (Table 2). In all cancers, except prostate and thyroid, the expression of *ETS1* and *SIX1* was significantly different. In kidney tumor tissues, *ETS1* expression exhibited a moderate inverse correlation with *SIX1* (Table 3).

### 3.5. ETS1 and SIX1 expression patterns in EMT-associated tumor contexts

#### 3.5.1. Context-dependent expression patterns of ETS1 and SIX1 across the TCGA cancer cohorts

**Figure 4:**
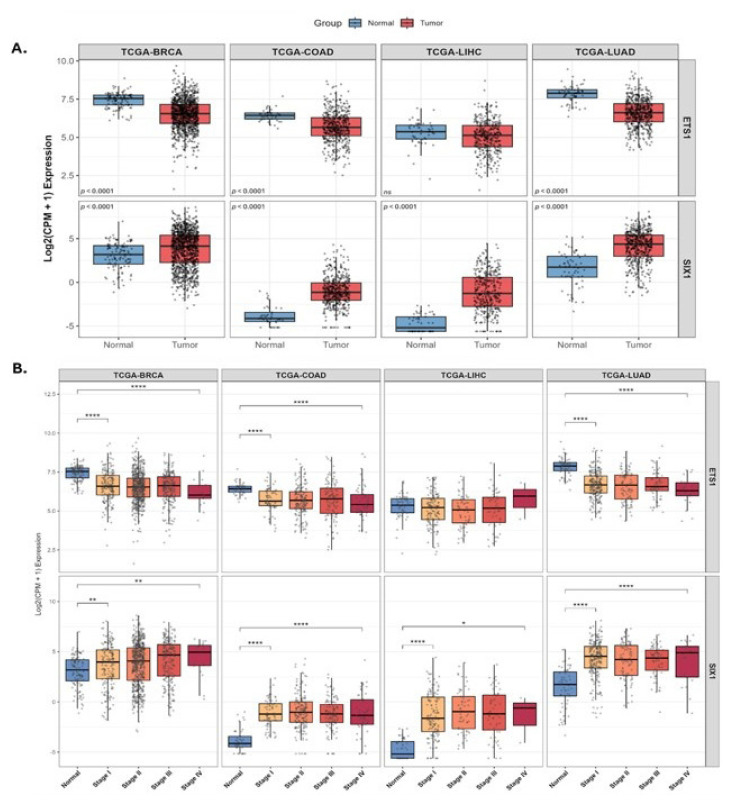
Differential expression of ETS1 and SIX1 across TCGA cancer types and pathological stages. **A**. Comparison of ETS1 (top row) and SIX1 (bottom row) gene expression between normal and tumor samples across the TCGA-BRCA, TCGA-COAD, TCGA-LIHC, and TCGA-LUAD cohorts (**** p.adj < 0.0001 and ns p.adj ≥ 0.05). (normal vs. tumor, pan-cancer) **B**. Distribution of ETS1 (top row) and SIX1 (bottom row) gene expression across normal samples and pathological stages (Stage I–IV) in the TCGA-BRCA, TCGA-COAD, TCGA-LIHC, and TCGA-LUAD cohorts (**** p.adj < 0.0001, *** p.adj < 0.001, ** p.adj < 0.01, * p.adj < 0.05, and ns p.adj ≥ 0.05).

Transcriptomic analyses of the TCGA cohorts demonstrated diverse and context-dependent expression patterns for ETS1 and SIX1. In the BRCA, COAD, and LUAD cohorts, ETS1 expression was decreased in tumor tissues relative to matched normal samples, whereas no significant change was noted in liver HCC (LIHC) (Figure 4A). In contrast, SIX1 expression was elevated in tumor samples across all four cancer types and showed higher intertumor variability, especially in the COAD and LIHC cohorts. Stage-stratified analyses demonstrated divergent expression patterns for ETS1 and SIX1. ETS1 expression exhibited relative stability or a modest decrease across pathological phases, while SIX1 expression increased with tumor stage in the BRCA and LUAD cohorts, with higher variability in the COAD and LIHC cohorts (Figure 4B).

#### 3.5.2. Prognostic associations of SIX1 and ETS1 expression across TCGA cancer cohorts

**Figure 5:**
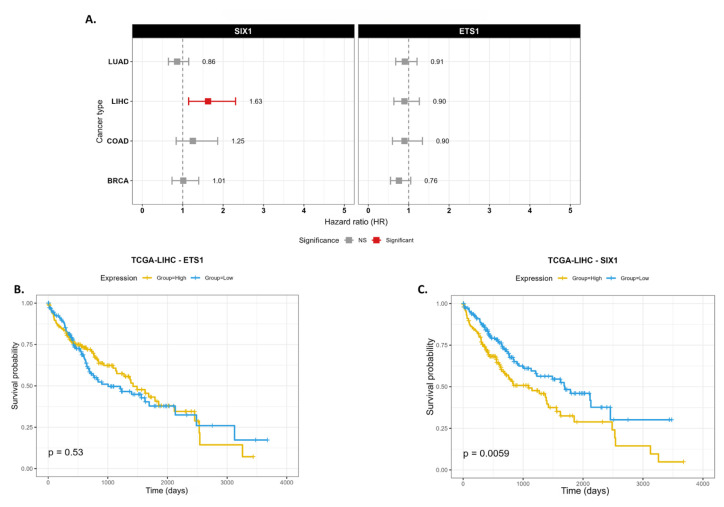
Pan-cancer and LIHC-specific survival associations of ETS1 and SIX1. **A**. Pan-cancer survival associations of *SIX1* and *ETS1* across TCGA cohorts (BRCA, COAD, LIHC, LUAD). Forest plots display hazard ratios (HRs) with 95% confidence intervals (horizontal bars) derived from Cox proportional hazards models for each cancer type. The vertical dashed line denotes HR = 1 (no effect). Statistically significant associations are highlighted in red, whereas non-significant results are shown in grey. In the LIHC cohort, *SIX1* expression was significantly associated with survival (HR = 1.63, 95% CI: 1.15–2.31, p = 0.0064), whereas *ETS1* showed no significant association (HR = 0.90, 95% CI: 0.63–1.26, p = 0.53). **B**. Patients from the TCGA-LIHC cohorts were classified into high and low *ETS1* expression groups, and the Kaplan–Meier survival curve is presented. Group differences were evaluated using the log-rank test (LIHC: p = 0.53). **C**. Patients from the TCGA-LIHC cohorts were classified into high and low *SIX1* expression groups, with Kaplan–Meier survival curves presented (LIHC: p = 0.0059).

Survival analyses were conducted in the LUAD, LIHC, COAD, and BRCA cohorts to assess the prognostic relevance of SIX1 and ETS1 expression. Cox regression analysis revealed that SIX1 expression was a significant prognostic indicator in the LIHC cohort, with elevated SIX1 levels correlating with a raised mortality risk (hazard ratio (HR) = 1.63, p < 0.05; Figure 5A). No significant correlation between SIX1 expression and overall survival was detected in the LUAD, COAD, or BRCA cohorts. In the same analysis, ETS1 expression did not demonstrate a significant correlation with overall survival across any of the assessed cancer types (Figure 5A). The Kaplan–Meier analysis consistently revealed no significant difference in survival between high and low ETS1 expression groups in the LIHC cohort (p = 0.53, Figure 5B). Conversely, individuals with elevated SIX1 expression demonstrated markedly reduced overall survival relative to those with low SIX1 expression in the LIHC group (p = 0.0059, Figure 5C).

#### 3.5.3. Context-dependent correlation patterns between ETS1 and SIX1 across the TCGA cohorts

**Figure 6:**
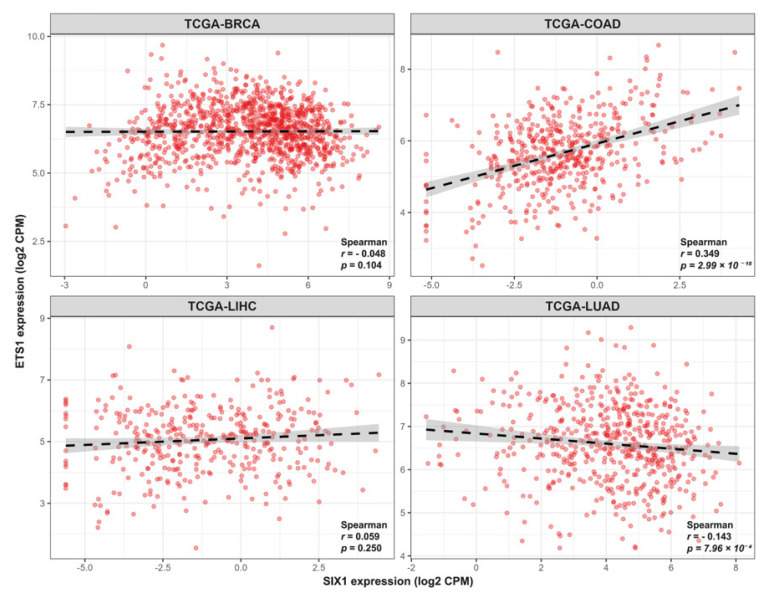
Correlation analysis of the association between SIX1 and ETS1 expression in tumor samples across the TCGA-BRCA, TCGA-COAD, TCGA-LIHC, and TCGA-LUAD cohorts.

Correlation analyses of ETS1 and SIX1 expression across TCGA cohorts demonstrated heterogeneous and context-dependent association patterns instead of a consistent regulatory relationship (Figure 6). In the BRCA cohort, there was no significant correlation between ETS1 and SIX1 expression (Spearman r = −0.048, p = 0.104). However, in the COAD cohort, a moderate positive correlation was found (r = 0.349, p = 2.99 × 10^−^^15^). Conversely, ETS1 and SIX1 expression exhibited no significant correlation in LIHC (r = 0.059, p = 0.250) and a weak inverse correlation in LUAD (r = −0.143, p = 7.96 × 10^−4^).

**Figure 7:**
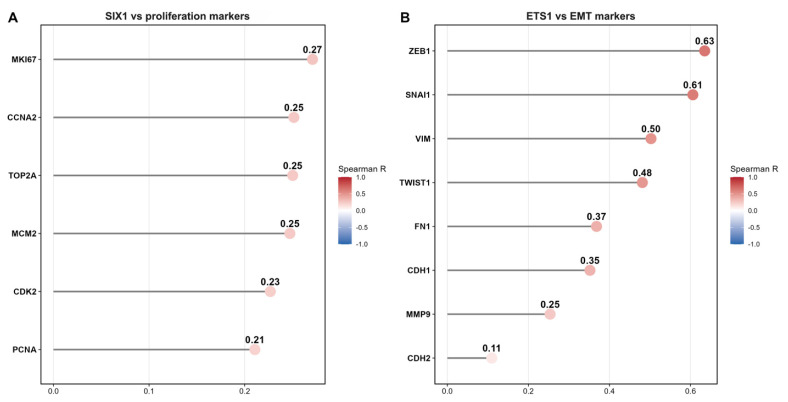
TCGA-LIHC: Correlation of SIX1 and ETS1 with proliferation and EMT markers. **A**. Spearman correlation between SIX1 and proliferation markers. **B**. Spearman correlation between ETS1 and EMT markers.

To further examine gene-level associations, correlation analyses were performed between ETS1 or SIX1 expression and established transcriptional markers of proliferation and EMT (Figure 7). SIX1 expression demonstrated modest yet consistent positive correlations with proliferation-associated genes, including *MKI67*, *CCNA2*, *TOP2A*, *MCM2*, *CDK2*, and *PCNA* (see Figure 7A).

Conversely, ETS1 expression demonstrated stronger positive correlations with canonical EMT-related genes, including *ZEB1*, *SNAI1*, *VIM*, *TWIST1*, and *FN1* (Figure 7B). These patterns indicate distinct transcriptional associations for ETS1 and SIX1 at the gene expression level.

In line with these findings, ETS1 silencing in liver tumor-derived endothelial-like SK-HEP-1 cells was associated with reduced mRNA expression of mesenchymal markers (*TWIST1*, *SNAI2*/*SLUG*, and *VIM*) and increased CDH1 (E-cadherin) expression, as assessed by qPCR (Figure S2).

#### 3.5.4. ETS1–SIX1 axis is associated with EMT, cell cycle progression, and immune-related pathways

GSEA of TCGA-LIHC samples stratified by ETS1 (Figure S3) or SIX1 (Figure S4) expression revealed distinct yet partially overlapping pathway signatures. High SIX1 expression was predominantly associated with enrichment of cell cycle–related pathways, whereas ETS1-high tumors showed marked enrichment of EMT and immune-related signaling programs, suggesting functional divergence within the ETS1–SIX1 axis in EMT-associated tumor states.

## 4. Discussion

Evidence was presented herein that demonstrates the interaction between ETS1 and SIX1 at the level of transcription. ChIP analyses and inverse expression patterns suggested that ETS1 is linked to the SIX1 promoter and may have a negative effect on its transcription. However, promoter-reporter assays are required to prove direct transcriptional repression. Further studies using luciferase-based reporter systems will be required in the future to confirm this regulatory mechanism.

Herein, endothelial-like SK-HEP-1 cells, a liver tumor-derived cell line with relatively high endogenous ETS1 expression (Yalim Camci et al., 2019), were used to identify a previously uncharacterized interaction within EMT-associated transcriptional networks. Although ETS1 has usually been described as a transcriptional activator, an increasing body of evidence suggests that its function depends on specific circumstances (Dittmer, 2003; Hollenhorst et al., 2011; Hunter et al, 2021). The finding observed in the current study may have been due to the presence of proteins that modify chromosome structure, such as histone deacetylases, in the SIX1 promoter region (Li et al., 2000). ETS1 works with several major signaling pathways, including TGF-β, mitogen-activated protein kinase/extracellular signal-regulated kinase, and phosphoinositide 3-kinase/protein kinase B/protein kinase B. This suggests that the output of ETS1 transcription is affected by signals from outside and inside the cell (Cowley and Graves, 2000; Plotnik et al., 2014). Thus, ETS1 helps to control SIX1, which helps to adjust the processes that control cell proliferation.

Examination of a human tumor cDNA panel demonstrated a predominantly inverse correlation between the ETS1 and SIX1 expression across several cancer types, indicating a context-dependent regulatory link. Pan-cancer investigations of the TCGA-BRCA, COAD, LIHC, and LUAD cohorts have consistently demonstrated differential expression patterns of ETS1 and SIX1 across normal and tumor tissues, as well as across pathological phases^1^. ETS1 expression was generally elevated in normal or well-differentiated samples, while SIX1 expression was increased in cancer tissues, particularly in advanced-stage LIHC cases. The slight correlations detected in bulk RNA-seq datasets may indicate intratumoral heterogeneity instead of a lack of biological association (Marusyk et al., 2012). Survival analysis indicated that ETS1 and SIX1 exhibit distinct behaviors. Studies indicate that elevated levels of SIX1 correlate with poorer prognoses for cancer patients. This link was particularly strong in a group of patients with a particular type of liver cancer. Nonetheless, ETS1 expression was not uniformly associated with overall survival, indicating that ETS1 may primarily function as a transcriptional regulator rather than a direct prognostic marker of patient outcomes.

The findings herein revealed that the *ETS1* and *SIX1* genes were linked to each other in different ways. *SIX1* showed weak but consistent correlations with markers related to proliferation, whereas *ETS1* was more closely associated with mesenchymal and EMT-related genes. Similar patterns have been reported across multiple cancer types, where *SIX1* predominantly supports proliferative and stemness-associated programs, while *ETS1* is more tightly coupled to EMT-related transcriptional states and cellular plasticity (Huang et al., 2022a; Guo et al., 2025; Wang et al., 2025). The findings suggest that the ETS1–SIX1 axis may contribute to the coordination of multiple cellular states rather than functioning as a single, dominant regulatory pathway, consistent with recent models of hybrid EMT and transcriptional adaptability (Ziman et al., 2025).

Taken together, these results support a model in which ETS1 acts as a context-dependent regulator of SIX1 within EMT-associated transcriptional networks. While further functional validation is required, the present study highlights the dynamic and plastic nature of ETS1–SIX1 regulation, offering a framework for understanding how transcriptional flexibility may contribute to tumor heterogeneity and progression.

## Supplementary data

Figure S1 Predicted ETS1 binding sites within the SIX1 promoter region

Figure S2 RT-qPCR analysis of EMT-associated marker expression following ETS1 knockdown in SK-HEP-1 cells.

Figure S3 GSEA of the TCGA-LIHC cohort stratified by ETS1 expression. **A**. Ridge plot showing the top four enriched biological processes. **B–E**. Enrichment plots for HALLMARK_EPITHELIAL_MESENCHYMAL_TRANSITION, HALLMARK_INFLAMMATORY_RESPONSE, HALLMARK_UV_RESPONSE_DN, and HALLMARK_ALLOGRAFT_REJECTION comparing high- and low-risk groups. GSEA, Gene Set Enrichment Analysis; NES, normalized enrichment score; FDR, false discovery rate.

Figure S4 GSEA of the TCGA-LIHC cohort stratified by SIX1 expression. A. Hallmark GSEA ridge plot showing the top four enriched biological processes. **B–E**. Enrichment plots for HALLMARK_E2F_TARGETS, HALLMARK_G2M_CHECKPOINT, HALLMARK_ALLOGRAFT_REJECTION, and HALLMARK_INTERFERON_GAMMA_RESPONSE comparing high- and low-risk groups. GSEA, Gene Set Enrichment Analysis; NES, normalized enrichment score; FDR, false discovery rate.

## Figures and Tables

**Table 1:** ChIP-qPCR primers.

Target gene	Primer	Sequence (5′–3′)	Amplicon size (bp)	Efficiency (%)
**ZEB1**	Forward	GGCGATGACCGCTCATTTA	100	96
	Reverse	GCCCTCTTCCTCGCGTA		
**SIX1-1**	Forward	GCAAGTCTGCAAGGGATGA	214	102
	Reverse	GAAAGGTGGGAAGCCAGTT		
**SIX1-1-2**	Forward	GGAATAAGCAAGAGTTTCAAGGATG	229	94
	Reverse	GCATATTGAGATGCAAGTGGG		

**Table 2:** Transcriptional profiling of ETS1 and SIX1 in human tumor cDNA arrays.

Cancer type	N	Gene	ΔΔCt	Differential
				expression
**Lung**	9	ETS1	−3.71	*** p < 0.001
		SIX1	2.4	
**Liver**	9	ETS1	−0.47	*** p < 0.001
		SIX1	9.2	
**Breast**	9	ETS1	2.59	*** p < 0.001
		SIX1	6.7	
**Colon**	8	ETS1	−1.32	** p < 0.01
		SIX1	5.4	
**Kidney**	9	ETS1	−1.48	** p < 0.01
		SIX1	2.6	
**Ovarian**	8	ETS1	−0.02	** p < 0.01
		SIX1	4.8	
**Prostate**	9	ETS1	0.19	NS
		SIX1	0.9	
**Thyroid**	9	ETS1	−0.9	NS
		SIX1	−1.25	

**Table 3:** Correlation analysis of SIX1 and ETS1 in tumor cDNA arrays.

Cancer type	Lung	Liver	Breast	Colon	Kidney	Ovarian	Prostate	Thyroid
***n***	9	9	9	9	9	9	9	9
***r value***	0.58	0.39	0.57	0.51	−0.49	0.63	0.18	0.42

n indicates the number of array data sets per cancer type. Correlations between *ETS1* and *SIX1* were analyzed using Pearson’s correlation test.

## Data Availability

The datasets analyzed during the current study are publicly available from the TCGA repository and were accessed via the Genomic Data Commons (GDC) portal^2^. No new large-scale sequencing datasets were generated or deposited in external repositories as part of this study; all experimental data supporting the findings are included within the article and its supplementary materials.

## Footnotes

1 UALCAN (2022). An update to the integrated cancer data analysis platform. [online]. Website: https://ualcan.path.uab.edu/ [accessed 30 June 2025].
