# Supplementary figures and images for: Context-dependent regulation of SIX1 by ETS1 within EMT-associated transcriptional networks
Source: Turk J Biol. 2026 Mar 23;50(3):185–96. doi: 10.55730/1300-0152.2801 (PMC13398590; doi:10.55730/1300-0152.2801)

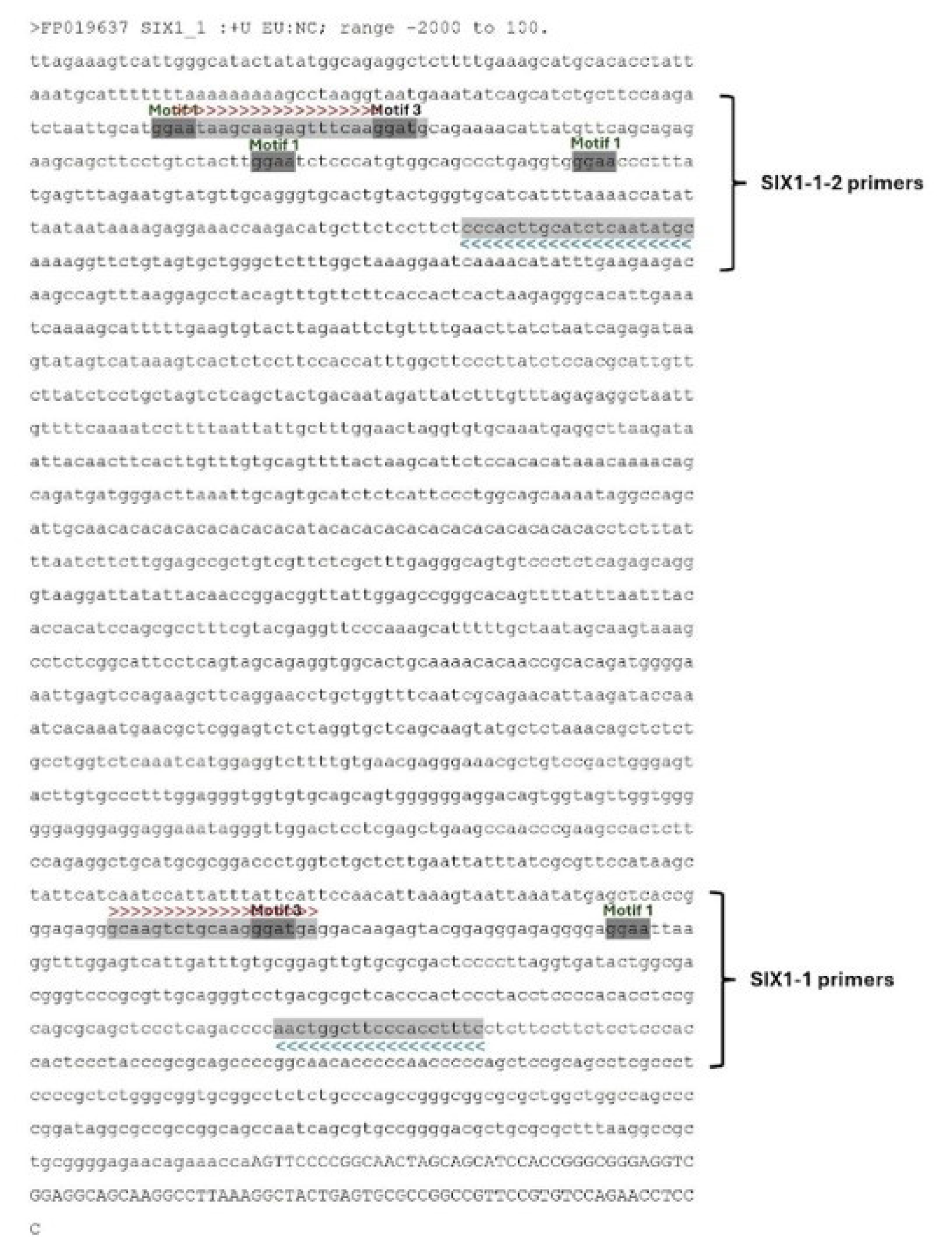

Supplement: Figure S1 — Predicted ETS1 binding sites within the SIX1 promoter region [file tjb-50-03-185s1.tif]

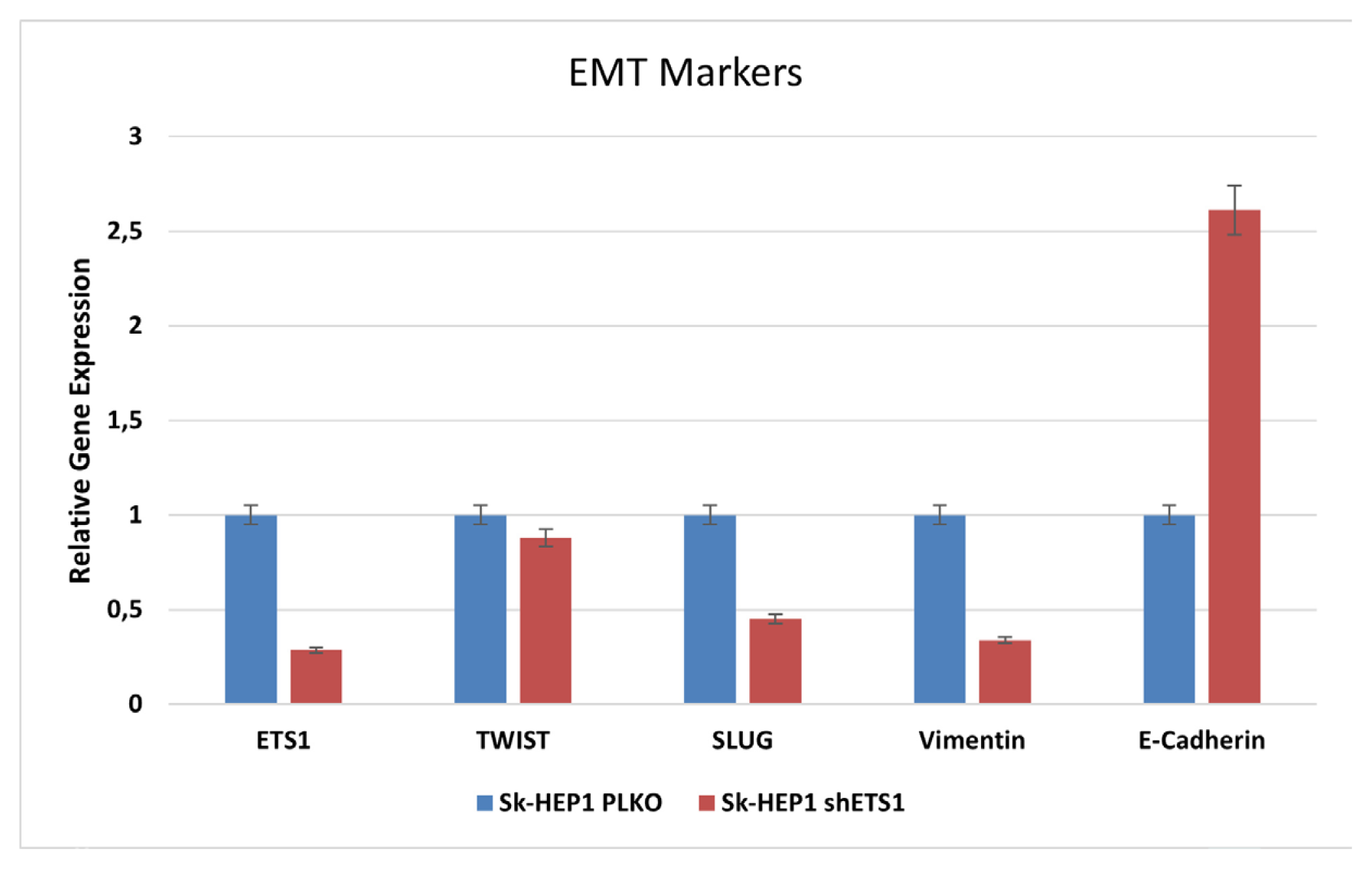

Supplement: Figure S2 — RT-qPCR analysis of EMT-associated marker expression following ETS1 knockdown in SK-HEP-1 cells. [file tjb-50-03-185s2.tif]

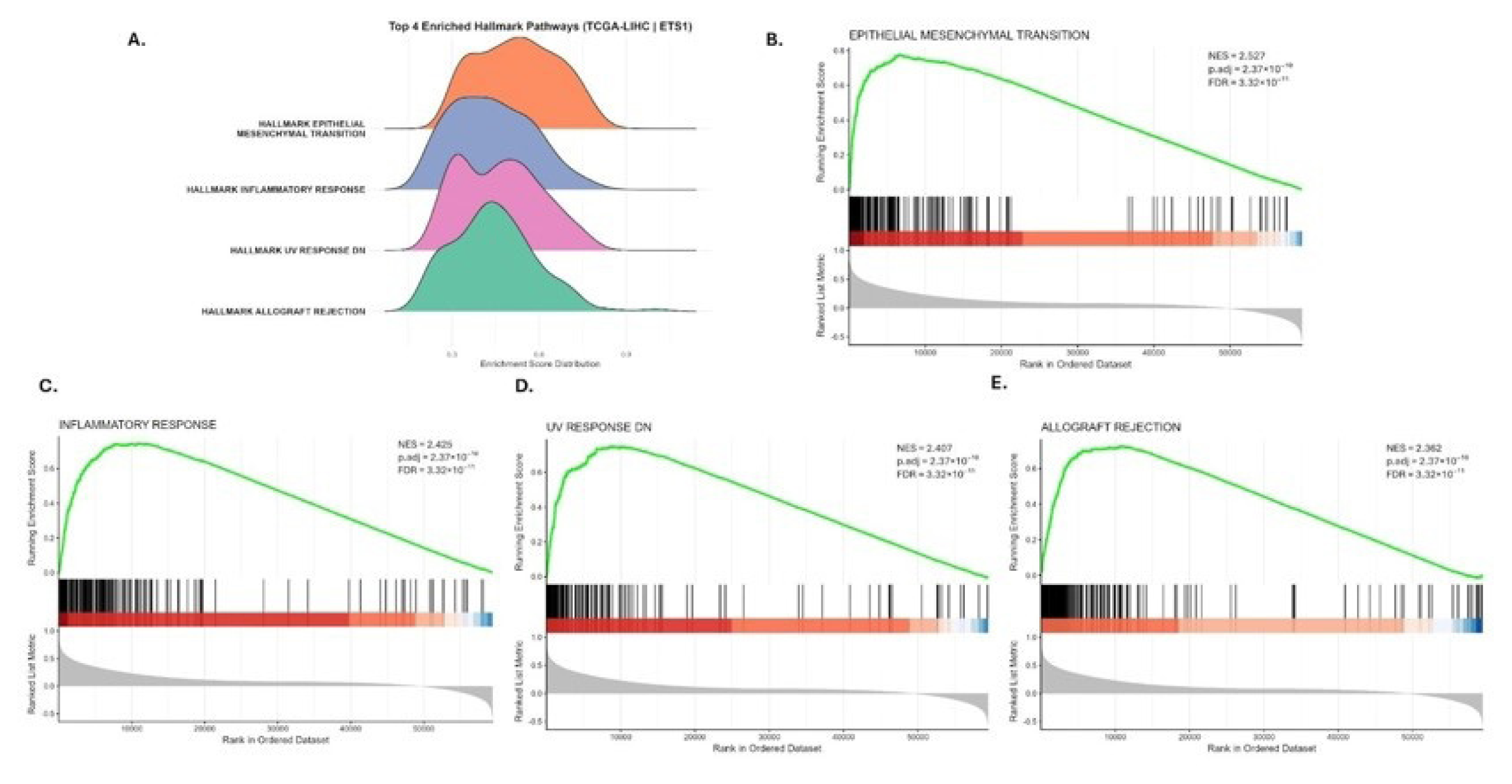

Supplement: Figure S3 — GSEA of the TCGA-LIHC cohort stratified by ETS1 expression. A. Ridge plot showing the top four enriched biological processes. B–E. Enrichment plots for HALLMARK_EPITHELIAL_MESENCHYMAL_TRANSITION, HALLMARK_INFLAMMATORY_RESPONSE, HALLMARK_UV_RESPONSE_DN, and HALLMARK_ALLOGRAFT_REJECTION comparing high- and low-risk groups. GSEA, Gene Set Enrichment Analysis; NES, normalized enrichment score; FDR, false discovery rate. [file tjb-50-03-185s3.tif]

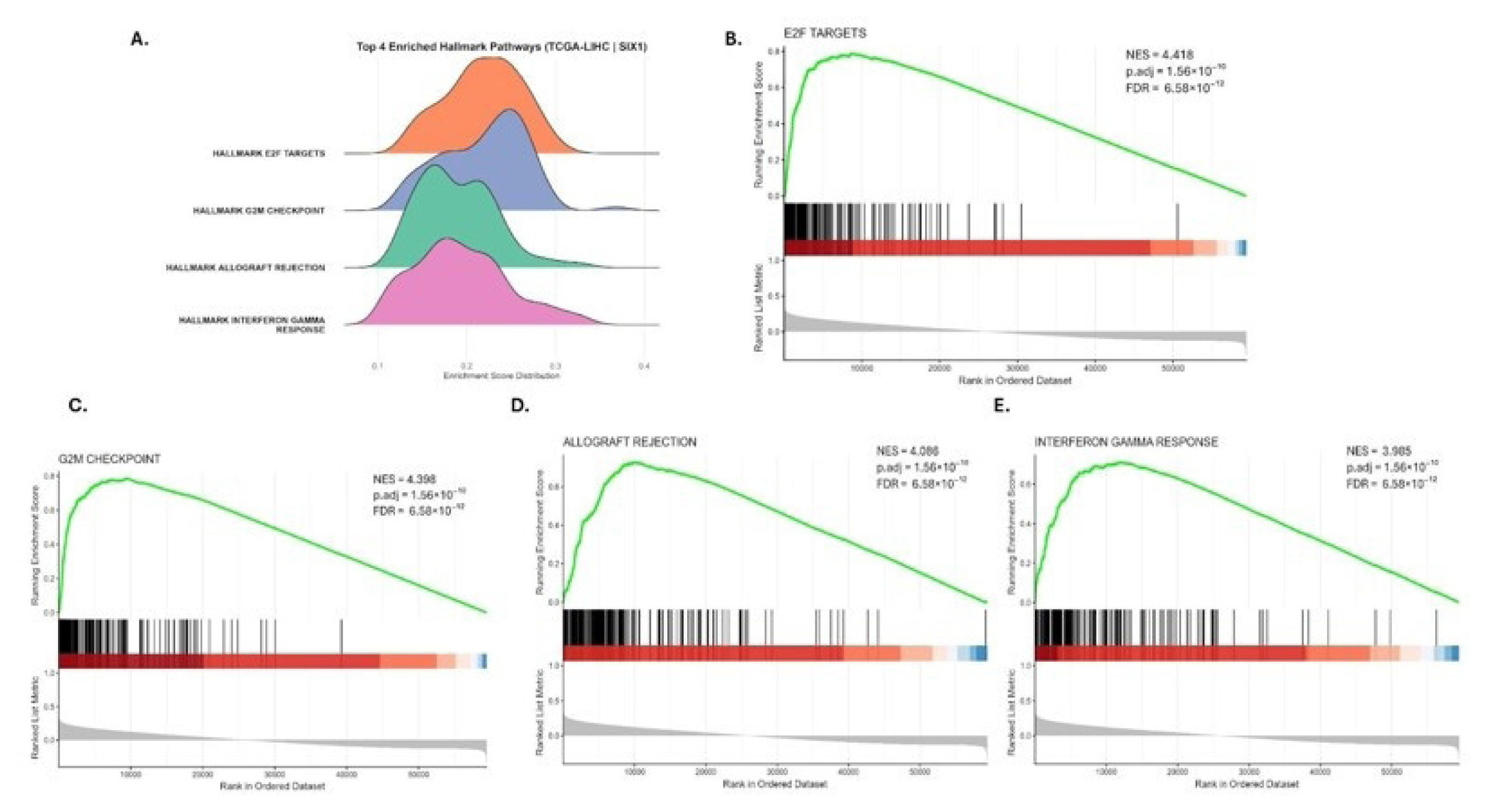

Supplement: Figure S4 — GSEA of the TCGA-LIHC cohort stratified by SIX1 expression. A. Hallmark GSEA ridge plot showing the top four enriched biological processes. B–E. Enrichment plots for HALLMARK_E2F_TARGETS, HALLMARK_G2M_CHECKPOINT, HALLMARK_ALLOGRAFT_REJECTION, and HALLMARK_INTERFERON_GAMMA_RESPONSE comparing high- and low-risk groups. GSEA, Gene Set Enrichment Analysis; NES, normalized enrichment score; FDR, false discovery rate. [file tjb-50-03-185s4.tif]
